# Corn Yield and Soil Nitrous Oxide Emission under Different Fertilizer and Soil Management: A Three-Year Field Experiment in Middle Tennessee

**DOI:** 10.1371/journal.pone.0125406

**Published:** 2015-04-29

**Authors:** Qi Deng, Dafeng Hui, Junming Wang, Stephen Iwuozo, Chih-Li Yu, Tigist Jima, David Smart, Chandra Reddy, Sam Dennis

**Affiliations:** 1 College of Agriculture, Human and Natural Sciences, Tennessee State University, Nashville, Tennessee 37209, United States of America; 2 Climate Science Section, Illinois State Water Survey, Prairie Research Institute, University of Illinois at Urbana-Champaign, Champaign, Illinois 61802, United States of America; 3 Department of Viticulture and Enology, University of California Davis, Davis, California 95616, United States of America; North Carolina State University, UNITED STATES

## Abstract

**Background:**

A three-year field experiment was conducted to examine the responses of corn yield and soil nitrous oxide (N_2_O) emission to various management practices in middle Tennessee.

**Methodology/Principal Findings:**

The management practices include no-tillage + regular applications of urea ammonium nitrate (NT-URAN); no-tillage + regular applications of URAN + denitrification inhibitor (NT-inhibitor); no-tillage + regular applications of URAN + biochar (NT-biochar); no-tillage + 20% applications of URAN + chicken litter (NT-litter), no-tillage + split applications of URAN (NT-split); and conventional tillage + regular applications of URAN as a control (CT-URAN). Fertilizer equivalent to 217 kg N ha^-1^ was applied to each of the experimental plots. Results showed that no-tillage (NT-URAN) significantly increased corn yield by 28% over the conventional tillage (CT-URAN) due to soil water conservation. The management practices significantly altered soil N_2_O emission, with the highest in the CT-URAN (0.48 mg N_2_O m^-2^ h^-1^) and the lowest in the NT-inhibitor (0.20 mg N_2_O m^-2^ h^-1^) and NT-biochar (0.16 mg N_2_O m^-2^ h^-1^) treatments. Significant exponential relationships between soil N_2_O emission and water filled pore space were revealed in all treatments. However, variations in soil N_2_O emission among the treatments were positively correlated with the moisture sensitivity of soil N_2_O emission that likely reflects an interactive effect between soil properties and WFPS.

**Conclusion/Significance:**

Our results indicated that improved fertilizer and soil management have the potential to maintain highly productive corn yield while reducing greenhouse gas emissions.

## Introduction

The United States (US) is the largest corn producer in the world, accounting for about 32% of the world's corn crop, with about 87.4 million ha in production in 2012 according to National Corn Grower’s Association (2013) [1]. To maintain high corn yield, large amounts of nitrogen (N) fertilizer are applied each year. About 5 million metric tons of N fertilizer were consumed in 2010 for corn production in the US, which accounted for 46% of the overall US consumption of N fertilizer [2]. However, nitrogen use efficiency (NUE) in corn production systems remains generally low: just 30%– 59% [3]. Much of the excess N applied to corn fields is leached into ground water as NO_3_^-^ or emitted to the atmosphere in the form of N_2_ and reactive N gases such as ammonia (NH_3_) and nitrous oxide (N_2_O) [4].

Nitrous oxide is one of the major greenhouse gases and has 310 times the radiative forcing potential of CO_2_ [5]. Agricultural soil is the primary source of N_2_O and the average annual N_2_O emission from corn fields in the US ranges from 1 to 3.2 tons CO_2_ equivalent per hectare [4]. The annual total N_2_O emission from US corn croplands is greater than 29.4 million tons of CO_2_ equivalent [6]. Therefore, knowledge of the trade-offs between soil N_2_O emission, fertilizer management practice, and corn yield is essential for the development of sustainable landscapes and best management practices in these agricultural systems.

Nitrous oxide emitted from soil is produced by microbial transformations of inorganic N, through a series of processes usually involving nitrification and denitrification [7]. The potential to produce and emit N_2_O increases as the amount of applied N fertilizer (availability of mineral N) increases in soil [8–11]. Due to the strong influence of N fertilizer on corn growth and yield, some emission of N_2_O from soil seems to be an unavoidable consequence of maintaining highly productive, economically sustainable corn cropland [12]. However, the relationships between N fertilizer and soil N_2_O emission may be more complex in practice [13]. Nitrogen often limits both crop growth and N_2_O production in agriculture, so that crops with sufficient N may better compete with microbes for soil inorganic-N [14, 15]. Thus, soil N_2_O production may be suppressed when crop NUE is enhanced in croplands [16, 17]. Soil N_2_O emission is also affected by several other factors such as crop type, soil type, soil oxygen supply, soil temperature, rainfall and water filled pore space (WFPS) [18–21]. Agricultural practices such as no-tillage and improved fertilizer N management may influence the emission of N_2_O by the modification of edaphic properties that affect soil microclimate [22–24]. A number of management practices have been proposed to enhance corn yield and reduce N_2_O emission, but they remain controversial because the related influencing factors are still misunderstood [17, 25–27]. So far, there have been very few studies conducted to simultaneously compare the response of corn yield and soil N_2_O emission under a range of proposed corn management practices [28, 29].

In this study, we conducted a three-year (2012–2014) field experiment in middle Tennessee to examine the responses of corn yield and soil N_2_O emission to a number of proposed sustainable management practices. In order to mimic the normal practice by farmers in middle Tennessee, we contrasted the treatment of conventional tillage + regular applications of urea ammonium nitrate with a range of N fertilizer practices under no-tillage conditions. All treatments received the same equivalent unit of N (217 kg N ha^-1^) but in different forms and application scenarios. The objectives of this study were: (1) to evaluate the effects of improved soil and fertilizer management practices on corn yield and soil N_2_O emission; (2) to detect any relationships between corn yield, soil N_2_O emission and changes in soil properties or microclimate under the different treatments.

## Materials and Methods

### Ethics statement

The study site is maintained by the College of Agriculture, Human and Natural Sciences, Tennessee State University. The location is within the Tennessee State University Agricultural Research Center. All necessary permits were obtained for the described field study. The field study did not involve endangered or protected species. Data will be made available upon request.

### Site description

This study was conducted at the Tennessee State University Agricultural Research Center (Latitude 36.12'N, Longitude 86.89'W, elevation 127.6 m) in Nashville, TN, USA. Climate in the region is a warm humid temperate climate (http://weatherspark.com/averages/29787/Nashville-Tennessee-United-States), with an average annual temperature of 15.1°C, and total annual precipitation of 1200 mm. The experimental site is a Talbott silt clay loam soil (Fine, mixed, semi-active, thermic Typic Hapludalfs; 25% sand, 55% silt, 20% clay) with a bulk density of 1.45 g cm^-3^, slightly acidic (pH = 5.97), low in both carbon (2.37 g kg^-1^) and nitrogen (0.14 g kg^-1^) in the upper 0–30 cm soil layer.

### Experimental Design

The experiment was laid out as a randomized complete block design with six replications in 2012–2013 and four replications in 2014 (two blocks were not used due to weed problems). Each block contained six plots. Six treatments were randomly assigned among the 6 plots within each block. Referring to the normal practice by farmers in middle Tennessee, we considered the treatment with conventional tillage + regular applications of aqueous urea ammonium nitrate (URAN-32-0-0 liquid N, 100%) as the control (CT-URAN). Improved fertilizer and soil management were used as the other five treatments, which included: no-tillage + regular applications of URAN (URAN-32-0-0 liquid N, 100%) (NT-URAN); no-tillage + regular applications of URAN (URAN-32-0-0 liquid N, 90%) + dicyandiamide (DCD) nitrification inhibitor with 67% N content (N, 10%) (NT-inhibitor); no-tillage + regular applications of URAN (URAN-32-0-0 liquid N, 100%) + woodchips biochar with density of 1.5–1.7 g cm^-3^ and with an application rate of 2.5 kg m^-2^ (NT-biochar); no-tillage + 20% applications of URAN (URAN-32-0-0 liquid N, 20%) + chicken litter with 4% N, 3% P and 4% K contents (N, 80%) (NT-litter); and no-tillage + split applications of URAN (URAN-32-0-0 liquid N, 100%) (NT-split). The total number of plots was 36. Plot size was 5.5 m in 7.0 m encompassing 12 rows of corn. Corn seeds (Roundup Ready BT Hybrid Corn, P1412 HR, Pioneer Hi-Bred International Inc., Johnston, IA) were sown on April 9 of 2012, April 25 of 2013 and May 14 of 2014, respectively. Rows were planted at 0.5 m intervals at a density of 100,500 seeds ha^-1^, resulting in 12 rows in 7.0 m direction per plot. Prior to planting, a non-selective herbicide (glyphosate) was sprayed to kill existing weeds in the plots. The CT plots were tilled to about 6 cm depth using a rotary harrow. At the planting, a total of 99 kg N ha^-1^ of chicken litter were applied to all the plots. During the experiment period, two applications of URAN fertilizer were applied on jointing stage (39 kg N ha^-1^) and heading stage (79 kg N ha^-1^) in the NT-URAN, NT-inhibitor, NT-biochar, NT-litter and CT-URAN plots, respectively. For the NT-split treatment, the applications of fertilizer-N were split by half, and therefore two additional fertilizer applications of 19.5 and 39.5 kg N ha^-1^ occurred (4 fertilizer applications in total). As a result, equivalent units of (217 kg N ha^-1^) were applied to all treatments, albeit in different forms to each experimental plot. Due to the severe drought in June of 2012, we irrigated all plots on June 14–15 at an equivalent amount of 50 mm water and June 30-July 2 at an equivalent amount of 90 mm, respectively.

### Soil N_2_O flux measurements

Gas samples were collected after rainfall event(s) or fertilizer applications or every two weeks during the growing season over three years using static chambers [30]. The static chamber was made of polyvinyl chloride (PVC) material and consisted of two parts: a soil ring without a top and bottom of 20 cm in diameter and 30 cm in height, and a removable cover of 20 cm in diameter and 6 cm in height. The ring was inserted directly into the soil about 25 cm below the soil surface leaving 5 cm above the soil surface, and the cover was placed on top during sampling and removed afterwards. A fan of 10 cm in diameter was installed on the top wall of each cover to create gentle turbulent mixing when the chamber was closed. A typical measurement started from 09:00–10:00 am and lasted for about 30 min. Gas samples (20 ml each) were generally collected at three time intervals (0, 15 and 30 minutes) using 20 ml plastic syringes. All gas samples were stored in sealed vacuum vials and then were transferred to analyze at the University of California, Davis within 96 h of collection for N_2_O concentrations using a gas chromatograph (Model GC-2014, Shimadzu Scientific Instruments, Columbia, MD) equipped with a ^63^Ni electron capture detector for quantifying N_2_O. Instantaneous soil N_2_O emission was calculated based on the rate of change in N_2_O concentration within the chamber, which was estimated as the slope of linear regression between concentration and time [31]. If linear regression has an R^2^ ≥ 0.9, it is accepted for instantaneous N_2_O flux rate estimate, if not, then a quadratic regression (R^2^ ≥ 0.9) is employed [32].

### Soil temperature and water filled pore space measurements

Soil temperature at 10 cm below the soil surface and volumetric soil moisture content of the top 10 cm soil depth layer were monitored in-situ when gas samples were collected. Soil temperature was measured using a digital thermometer probe (Taylor Thermometers USA, model number Taylor 9842). Volumetric soil moisture content was measured using a soil moisture probe (Extech Instruments USA, model number MO750). Water filled pore space (WFPS, %) was calculated from the equation: WFPS = SWC/(1-BD/PD), where SWC is the volumetric soil moisture (θv), BD is the bulk density (Mg m^-3^), and PD is the particle density (2.65 Mg m^-3^). Climatic data (rainfall and air temperature) were obtained from a weather station at the experimental site.

### Soil inorganic nitrogen measurements

Soil from each plot was sampled using a 5-cm diameter stainless steel soil probe. Samples were taken eleven times in 2012 from a depth of 0–30 cm at five random locations within each plot. At each time, a total of 6 composited samples were collected to represent each treatment, with a grand total of 36 mixed samples across all six treatments. Soil samples were air-dried and sieved to pass thru a 2-mm mesh. Soil NH_4_^+^-N and NO_3_^-^-N were extracted with a 2 M potassium chloride (KCl) solution (soil:solution, 1:5) and then filtered through a 0.45μm filter. The extracted solutions were measured via colorimetric techniques at 645 nm and 420 nm to determine NH_4_^+^-N and NO_3_^-^-N concentrations, respectively. All measurements were performed in the Soil, Water and Forage Analytical Laboratory (SWFAL, a USDA certified lab) at the Oklahoma State University, Stillwater, OK.

### Corn yield measurements

In each year of harvest, ten corn plants in each plot were selected to quantify grain yield in each year. Corn ears were removed by hand at harvest, then shelled and dried to a constant level. After harvest, most of crop residues were removed out of plots in each year.

### Statistical analysis

All data analyses were carried out with the SPSS software Version 13.0 (SPSS Inc., Chicago, IL). Repeated-measures Analysis of Variance (ANOVA) was used to determine the statistical significance of treatment, sampling year and their interactive effects on soil temperature, WFPS, and soil N_2_O emission in the corn fields. Multiple comparisons (Least Significant Difference, LSD method) were conducted if significant effects of treatment or sampling time were found. Two-way ANOVA with LSD test was used to determine the statistical significance of treatment, sampling year and their interactive effects on corn yield. The relationship between soil N_2_O emission rate (NE) and water filled pore space (WFPS) in each treatment was developed using the exponential model [NE = NE_0_×exp(b×WFPS), where parameter NE_0_ is basal soil N_2_O emission when WFPS = 0, and b is related to soil water sensitivity]. The *t*-test was used to determine the difference in the soil moisture sensitivity (b value) of N_2_O emission between the treatments.

## Results

### Microclimates and soil properties

Soil temperature ranged from 17.1°C to 35.4°C, with no significant difference among the treatments (Tables 1 and 2). WFPS ranged from 12.7 to 53.8%. The CT-URAN and NT-biochar treatments had significantly lower WFPS than those in other treatments (Tables 1 and 2). Seasonal patterns of soil temperature and WFPS were consistent with air temperature and rainfall, respectively. There was a severe drought in June 2012 with only 6.6 mm rainfall. Mean soil NH_4_^+^-N concentration was the highest in the NT-biochar treatment (26.77 mg kg^-1^), and the lowest in the NT-litter treatment (16.65 mg kg^-1^). Mean soil NO_3_^-^-N concentration was highest in the CT-URAN treatment (69.45 mg kg^-1^), and lowest in the NT-split treatment (45.78 mg kg^-1^).

**Table 1:** Significance of the effects of treatment, sampling year and their interactions on soil temperature, water filled pore space (WFPS), N_2_O emission, and corn yield based on ANOVAs.

Source	Soil temperature	WFPS	N_2_O emission	Yield
Treatment	0.10	3.44**	3.11*	3.04*
Year	36.92**	30.48**	5.35**	21.77**
Treatment×year	0.49	0.69	0.36	0.15

Numbers are F-values. Asterisks indicate the level of significance (* *p*<0.05, ** *p*<0.01).

**Table 2:** Soil temperature (°C), water filled pore space (WFPS; %), and inorganic-N (NH4+-N and NO_3_^-^-N for 2012 only; mg kg^-1^) under different tillage and fertilizer treatments in the corn fields at the Tennessee State University Agricultural Research Center in the southeastern United States.

Treatment	Soil temperature	WFPS	NH_4_^+^-N	NO_3_^-^-N
NT-URAN	28.70±0.33^a^	41.16±0.95^a^	5.02±0.42^bc^	13.01±1.04^bc^
NT-inhibitor	28.69±0.30^a^	40.46±0.99^ab^	5.57±0.35^abc^	15.60±1.15^ab^
NT-biochar	28.48±0.29^a^	38.42±0.93^bc^	6.89±0.82^a^	15.43±1.37^ab^
NT-litter	28.40±0.30^a^	41.12±0.87^a^	4.29±0.31^c^	14.30±1.00^b^
NT-split	28.82±0.30^a^	41.31±0.87^a^	4.79±0.31^c^	11.79±0.86^c^
CT-URAN	28.58±0.29^a^	36.26±0.85^c^	6.23±0.48^ab^	17.88±1.56^a^

Different letters in the same column indicate statistical significance at *α* = 0.05. NT-URAN = no-tillage + regular applications of URAN; NT-inhibitor = no-tillage + regular applications of URAN + nitrification inhibitor; NT-biochar = no-tillage + regular applications of URAN + biochar; NT-litter = no-tillage + chicken litter; NT-split = no-tillage + split applications of URAN; and CT-URAN = conventional tillage + regular applications of URAN.

### Corn yield and Soil N_2_O emission

**Fig 1:**
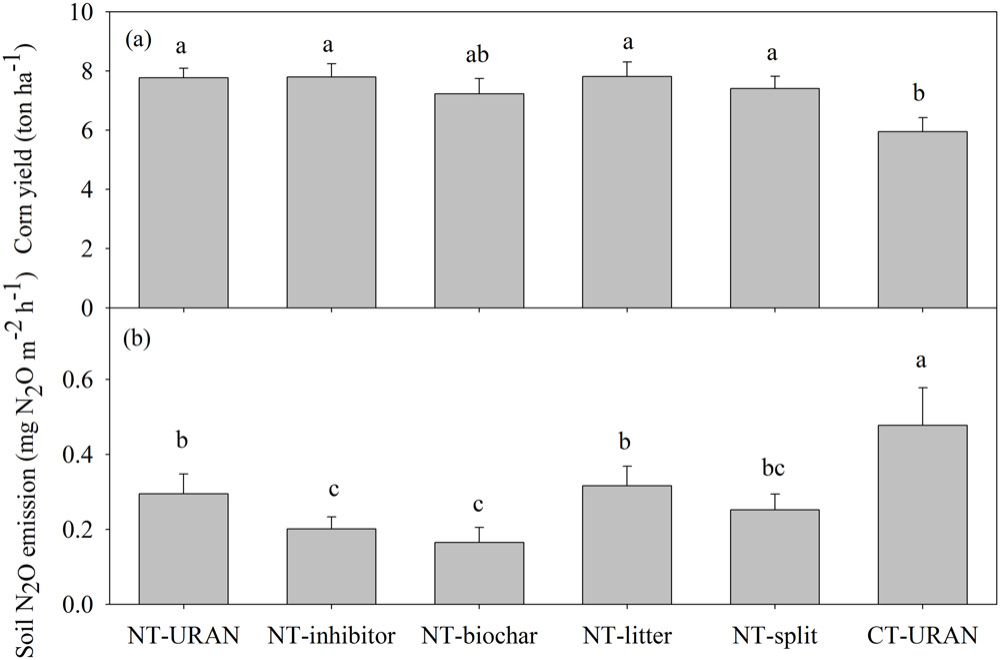
The average values of corn yield (a) and soil N_2_O emission (b) under different tillage and fertilizer treatments in the corn fields. Different letters over the bars indicate statistically significant differences at *α* = 0.05.

Corn yield was generally lower in the drought year of 2012 than the other two years (*p*<0.05). The management practices had significant effects on corn yield (Table 1). Specifically, corn yield was significantly affected by no-tillage with a higher value in the NT-URAN treatment (7.77 tones ha^-1^) than in the CT-URAN treatment (5.94 tones ha^-1^) across all three years (*p*<0.05, Fig 1a). No significant difference of corn yield was detected among the other fertilizer and soil management practices (Fig 1a). Corn yields were 7.79, 7.23, 7.81 and 7.41 tones ha^-1^ in the NT-inhibitor, NT-biochar, NT-litter and NT-split treatments, respectively.

Soil N_2_O emission was also significantly affected by improved fertilizer and soil management practices (Table 1). Within the experimental period, the highest N_2_O release from soil occurred in the CT-URAN (0.48 mg N_2_O m^-2^ h^-1^), which was significantly higher than those in the NT-URAN (0.29 mg N_2_O m^-2^ h^-1^) and NT-litter (0.32 mg N_2_O m^-2^ h^-1^) treatments, and much higher than those in the NT-inhibitor (0.20 mg N_2_O m^-2^ h^-1^), NT-biochar (0.16 mg N_2_O m^-2^ h^-1^), and NT-split (0.25 mg N_2_O m^-2^ h^-1^) treatments (Fig 1b).

### Controls on corn yield and soil N_2_O emission

**Fig 2:**
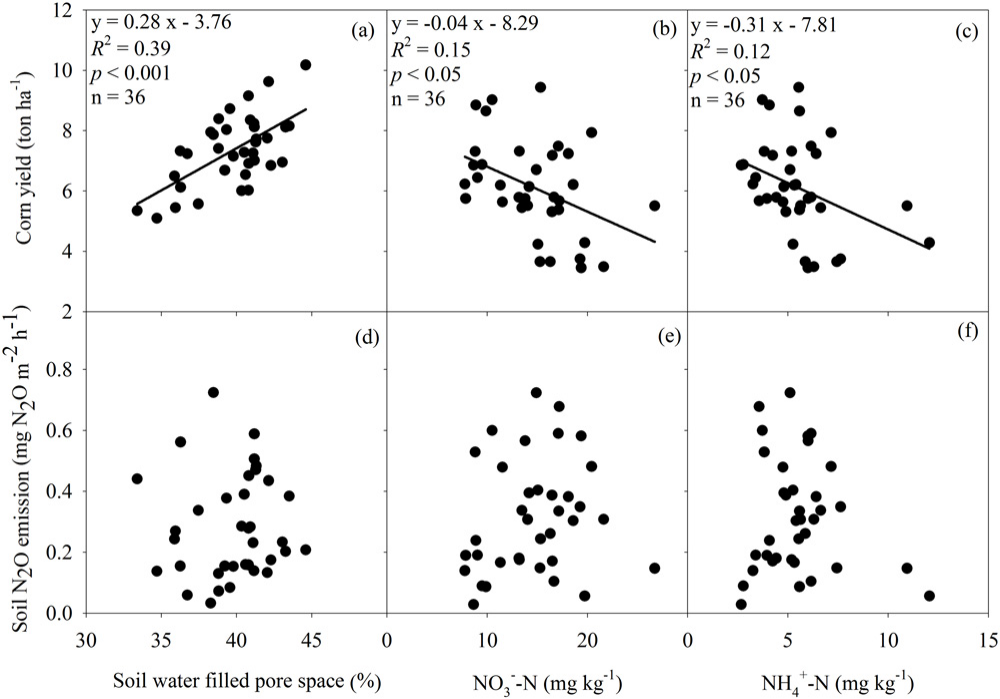
Relationships of corn yield (a, b, c) and soil N_2_O emission (d, e, f) with water filled pore space and soil inorganic-N (NH4+-N and NO_3_^-^-N, for 2012 only), respectively across all the plots.

**Fig 3:**
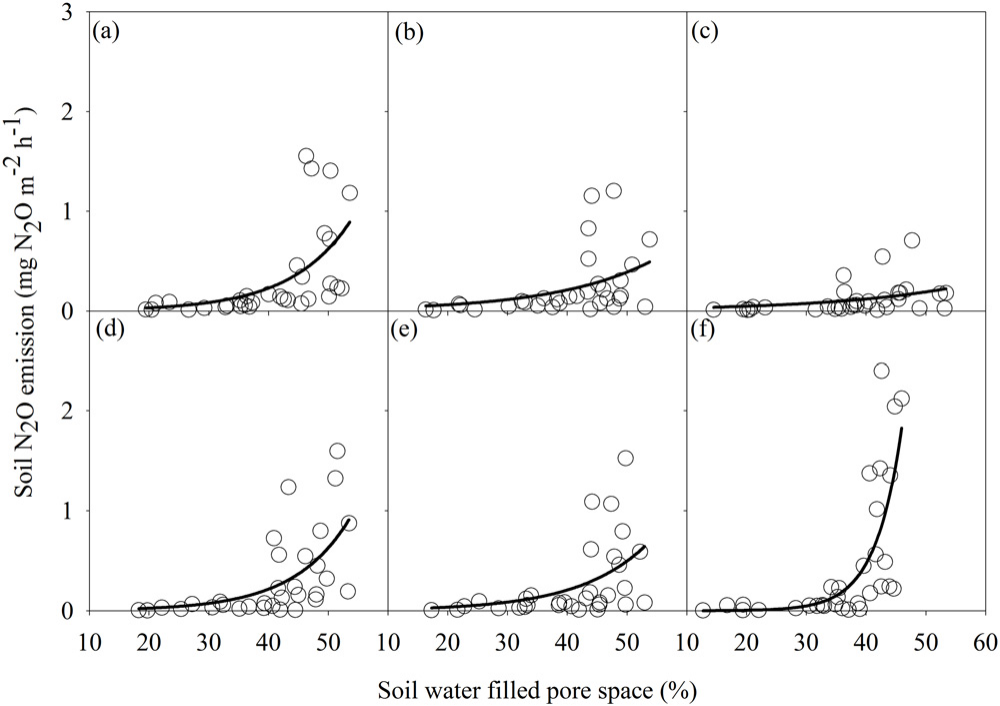
Relationships between soil N_2_O emission (NE, mg N_2_O m^-2^ h^-1^) and water filled pore space (WFPS, %) under different tillage and fertilizer treatments (a, b, c, d, e, f represent the NT-URAN, NT-inhibitor, NT-biochar, NT-litter, NT-split, CT-URAN treatments) in the corn fields. The equations are listed in Table 3.

**Fig 4:**
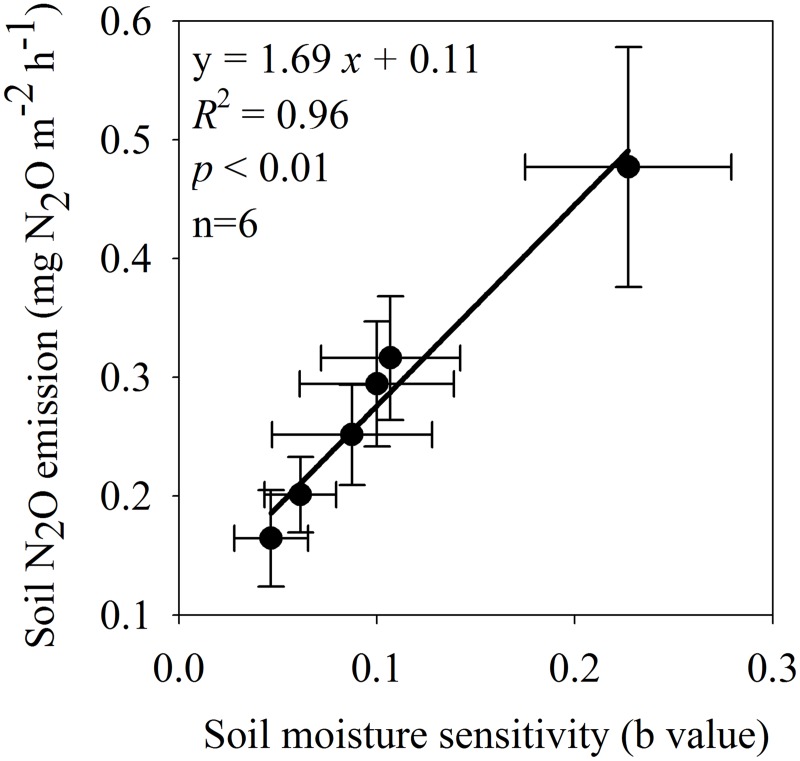
Relationships between soil N_2_O emission and its moisture sensitivity (b value) across all the treatments.

Corn yield was positively correlated with WFPS across all plots (Fig 2a). In contrast, corn yield was negatively correlated with inorganic-N (NH_4_^+^-N and NO_3_^-^-N) in all treatments (Fig 2b and 2c). No significant relationship between soil N_2_O emission rate and WFPS or inorganic-N was detected across all plots (Fig 2d–2f). There were significant exponential relationships between soil N_2_O emission and WFPS in each treatment (Fig 3). The management practices significantly altered the moisture sensitivities (b values) of soil N_2_O emission, with the highest in the CT-URAN (b = 0.23), relatively low in the NT-inhibitor (b = 0.06), NT-biochar (b = 0.05) and NT-split (b = 0.08), and intermediate in the NT-URAN (b = 0.10) and NT-litter (b = 0.11) treatments (Table 3). Soil N_2_O emission was positively correlated with its moisture sensitivity (b value) among the six treatments (Fig 4).

**Table 3:** Relationships between soil N_2_O emission rate (NE, mg N_2_O m^-2^ h^-1^) and water filled pore space (WFPS, %) under different tillage and fertilizer treatments in the corn fields at the Tennessee State University Agricultural Research Center in the southeastern US.

Treatment	NE_0_	b	*R* ^2^
NT-URAN	0.0042±0.0081	0.1000±0.0390^AB^	0.30**
NT-inhibitor	0.0181±0.0295	0.0614±0.0347^B^	0.22**
NT-biochar	0.0186±0.0236	0.0466±0.0281^B^	0.13*
NT-litter	0.0030±0.0052	0.1069±0.0351^AB^	0.43**
NT-split	0.0063±0.0128	0.0875±0.0405^B^	0.22**
CT-URAN	0.0001±0.0001	0.2270±0.0521^A^	0.47**

An exponential equation [NE = NE_0_×exp(b×WFPS) is applied, where parameter NE_0_ is basal soil N_2_O emission when WFPS = 0, and b is related to soil water sensitivity] (parameter estimate ± standard error).

Different capital letters in the same column indicate statistical significance at *α* = 0.05. * and ** indicate significant at α = 0.05, and 0.01 levels, respectively. NT-URAN = no-tillage + regular applications of URAN; NT-inhibitor = no-tillage + regular applications of URAN + nitrification inhibitor; NT-biochar = no-tillage + regular applications of URAN + biochar; NT-litter = no-tillage + chicken litter; NT-split = no-tillage + split applications of URAN; and CT-URAN = conventional tillage + regular applications of URAN.

## Discussion

The findings from our three-year field experiment provide insights into the effects of various management practices on corn yield and N_2_O emission in southeastern US and may have significant implications for sustainable agriculture and climate change mitigation. First, we found that no-tillage (NT-URAN) significantly increased corn yield by 28% over the conventional tillage (CT-URAN) probably due to soil water conservation. Second, we detected significant exponential relationships between soil N_2_O emission and WFPS in this study. The alternative fertilizer sources and improved soil management could decrease N_2_O emissions by reducing its moisture sensitivity. These findings are useful for better optimizing agricultural practices to maintain highly productive corn yield while reducing greenhouse gas emissions in the southeastern US.

### Corn yield

Our results demonstrated that corn yield was significantly affected by no-tillage, with a higher value in the NT-URAN treatment (7.77 tones ha^-1^) than in the CT-URAN treatment (5.94 tones ha^-1^) (*p*<0.05, Fig 1a). The corn yields presented here fall in the range of corn yield (1.0–17.5 ton ha^-1^) reported by a number of similar studies worldwide [33]. Our results for the no-tillage effect were different from DeFelice et al. [34] who reviewed 687 site-years of data in the US and Canada, and found that the average difference in corn yields between tillage and no-tillage treatments was negligible. In their analysis, they compared corn yields using total or region-level means. The total or mean yields were calculated from different climate, soil types and agricultural practices such as timing of planting, fertilizer placement, fertilizer rates, planting populations, irrigation and weed control methods. It is quite likely that the mean status of soil condition and management practices for tilled corn fields was different from that for no-tillage corn fields.

Our results were also different from a recent study that reported an approximate 30% decrease in corn yield in no-tillage corn fields compared to tilled corn fields in the cool humid region of Northeast China [35]. Temperature was often a dominant factor influencing agricultural production in that region. Consequently, they explained that a shift in soil temperature contributed to the difference in corn yield between no-tillage and tilled corn fields. Our study site in the southeastern US has a warm humid temperate climate with an average annual temperature of 15.1°C, and daily mean temperature ranged from 14.1°C to 32.7°C during this study. Thus, temperature should not be a dominant factor influencing agricultural production in this region. Indeed, we did not find any significant difference in soil temperature between all the treatments (Table 2). In contrast, crop yield in this study likely benefited from water conservation under no-tillage treatments even in this humid climatic region, as we often face short-term droughts in the summer months like that observed in June 2012 when only 6.6 mm of rainfall felt. Higher WFPS in the no-tillage compared to the tillage corn fields could contribute to higher yield in the NT treatment (Table 2). A similar result was reported in a 6-yr corn study, reporting that no-tillage conserved soil water and enhanced yield [26]. The positive relationships detected between corn yield and WFPS across all treatments further suggested that the no-tillage and improved fertilizer management influence corn yield mainly though a modification of WFPS, probably interactively due to the presence of drought events. Our results supported Franzluebbers’s [36] conclusion that soil water is likely a significant factor limiting crop production in the southeastern US.

Fertilizer management did not significantly change corn yield, perhaps due to the large amount of N fertilizer (217 kg N ha^-1^) applied to each treatment. The applied N fertilizer likely exceeded the demand of corn growth, so corn production was not subject to N limitation. A long-term corn (*Zea mays* L.) cropping system study has also shown that corn yields increase with N fertilizer from 0 to 101 kg N ha^-1^ and then level off at higher applications of N [14]. The maximum yield in that study varies from 5.4 to 9.0 ton ha^-1^ depending on the climate conditions. In addition, we detected a negative rather than positive relationship between corn yield and extractable soil N (NH_4_^+^-N and NO_3_^-^-N). It is probably that low corn yield or NUE consumed less N and let more inorganic N remaining in the soil [3]. Other ecological processes such as leaching and microbial assimilation may also contribute to the results for extractable soil N [4].

### Soil N_2_O emission

Previous studies have indicated that the level of N fertilizer application is one of the main factors influencing soil N_2_O emission [11, 14, 37]. The large amount of N fertilizer applied in our study might have led to high emission of N_2_O from corn fields (Fig 1b). The average rate of N_2_O release from soil in our study ranged from 0.16 to 0.48 mg N_2_O m^-2^ h^-1^, which was generally higher than those from a recent analysis of global mean level in N_2_O emissions (0.16 mg N_2_O m^-2^ h^-1^) where global average level of N fertilizer was only 152 kg N ha^-1^ [33]. Similarly, with less application of fertilizer to corn fields [38–40], less N_2_O emissions were reported. When equal amounts of N fertilizer were applied in an adjacent commercial corn field in Nolensville [41], we found similar N_2_O emission in the NT-URAN treatment, although N_2_O fluxes were measured using the eddy-covariance technique.

In addition to the level of N fertilizer application, the improved fertilizer and soil management significantly affected soil N_2_O emission compared with the CT-URAN treatment, where relatively low emissions were observed from the NT-URAN and NT-litter treatments and much lower emissions were observed in the NT-inhibitor, NT-biochar and NT-split treatments (Fig 1b). It is well-known that DCD as a nitrification inhibitor reduced the rate of nitrification, and resulted in a low emission of N_2_O [21, 42]. Significant differences of NH_4_^+^-N and NO_3_^-^-N between the treatments were detected perhaps due to shifts in corn yield and NUE (Fig 2b and 2c). However, variations in soil inorganic-N did not explain the difference of soil N_2_O emission among these treatments (Fig 2e and 2f). This indicated that soil inorganic-N probably interacted with other soil properties or microclimate to influence soil N_2_O emission under the different treatments [22, 43].

Rainfall or WFPS is one of the major factors driving soil N_2_O emission. Several studies have confirmed that there are connections between increased soil N_2_O emissions and rainfall or WFPS [19–21, 44]. We detected significant exponential relationships between soil N_2_O emission and WFPS in all treatments (Fig 3). Although there were large differences of WFPS among the treatments, however, no significant relationship between soil N_2_O emission and WFPS was detected across all treatments (Fig 2d–2f). Interestingly, we found that the variation in soil N_2_O emission was positively correlated with its moisture sensitivity (b value) among treatments (Fig 4). This indicated that improved fertilizer and soil management reduced soil N_2_O emission mainly though decreasing its moisture sensitivity that likely reflects a synthesized impact of soil properties.

Several soil properties might contribute to the shift of soil moisture sensitivity between treatments (Table 3) observed in this study. First, N_2_O emitted from soil is produced by bacterial transformations of inorganic-N, particularly of NO_3_^-^-N [8–10]. Concentrations of both NH_4_^+^-N and NO_3_^-^-N were highest in the CT-URAN treatment (Table 2), probably due to the reduced corn growth and in turn lower NUE. The higher extractable soil mineral-N provides more substrate for bacterial nitrification and denitrification [7, 14], and hence led to larger N_2_O emission during the periods of high WFPS (Table 3). In contrast, extractable NH_4_^+^-N and NO_3_^-^-N concentrations were low in the NT-split treatment, leading to lower moisture sensitivity of N_2_O emission (Table 3). This indicated that decreasing synthetic fertilization rate or reasonably arranging fertilization time could enhance corn NUE, and ultimately reduce N_2_O emission. Second, nitrification inhibitors such as DCD have been shown to reduce emissions of N_2_O directly by reducing the rate of NH_4_^+^-N oxidation to NO_3_^-^-N associated with nitrification, or indirectly by reducing microbial activity [21, 42]. Consequently, the NT-inhibitor treatment significantly decreased N_2_O emission and its moisture sensitivity (Table 3; Fig 1b), even though soil mineral-N availability was high (Table 2). However, it is notable that increasing inorganic N possibly results in subsequent losses by leaching and denitrification after DCD has been degraded [45, 46]. Similarly, biochar has high total porosity [47, 48] and can both retain water in small pores and maintain aerobic conditions in soil by letting water flow through the larger pores after heavy rain from topsoil to deeper soil layers [49, 50]. Thus, the NT-biochar treatment significantly decreased N_2_O emission and its soil moisture sensitivity (Table 3; Fig 1b). Our results differed from a recent study that reported biochar addition affected N_2_O emission via soil moisture and crop N uptake [17]. The difference was probably due to different biochar materials, crop types and fertilizer levels supplied in our as compared to their study [24].

## Conclusions

This study demonstrated that no-tillage could significantly reduce soil N_2_O emission while improving corn yield in the southeastern US. Split-application of fertilizer, nitrification inhibitor and bichar addition did not change corn yield, but did significantly reduce N_2_O emissions. Changes in corn yield were positively related to WFPS, indicating that any management that saves water could probably improve corn yield in this region. The seasonal dynamic of N_2_O emission from soil showed significant exponential relationships with WFPS in each treatment. However, alternative fertilizer sources (regular, spilt and chicken litter applications) and soil management (e.g., no-tillage, inhibitor and biochar) can decrease N_2_O emissions by reducing its moisture sensitivity of N_2_O emission rather than WFPS, This likely indicates an existing interaction of soil properties and WFPS impacts on N_2_O emissions from corn fields under various management practices. Overall, this study provides evidence that alternative soil and fertilizer management have the potential to maintain highly productive corn yield while reducing greenhouse gas emissions in the southeastern US.

## References

[1] National Corn Grower's Association (NCGA) (2013) Report. N.p., 11 Feb. 2013. Available: http://www.ncga.com/upload/files/documents/pdf/WOC%202013.pdf

[2] USDA Economic Research Service (2013) Available: http://www.ers.usda.gov/data-products/fertilizer-use-and-price.aspx#26720

[3] Halvorson AD, Schweissing FC, Bartolo ME, Reule CA (2005) Corn response to nitrogen fertilization in a soil with high residual nitrogen. Agron J 97: 1222–1229.

[4] Ogle SM, Del Grosso SJ, Adler PR, Parton WJ (2008) Soil nitrous oxide emissions with crop production for biofuel: implications for greenhouse gas mitigation In Outlaw JL, Ernstes DP, editors. The Lifecycle Carbon Footprint of Biofuels. Farm Foundation, pp. 11–18.

[5] CRS (2010) Nitrous oxide from agricultural sources: potential role in greenhouse gas emission reduction and ozone recovery. Congressional Research Service, 7–5700. R40874.

[6] EPA (2009) Major crops grown in the United States Ag101. U.S. Environmental Protection Agency Available: http://www.epa.gov/oecaagct/ag101/cropmajor.html

[7] Davidson EA, Swank WT (1986) Environmental parameters regulating gaseous nitrogen losses from two forested ecosystems via nitrification and denitrification. Appl Environ Microb 52: 1287–1292. 16347234 10.1128/aem.52.6.1287-1292.1986 PMC239223

[8] Bouwman AF (1996) Direct emission of nitrous oxide from agricultural soils. Nutr Cycl Agroecosyst 46: 53–70.

[9] Bouwman AF, Boumans L, Batjes NH (2002) Emissions of N_2_O and NO from fertilized fields: summary of available measurement data. Global Biogeochem Cycles 16: 1–13.

[10] Ma BL, Wu TY, Tremblay N, Deen W, Morrison MJ, Mclaughlin NB, et al (2010) Nitrous oxide fluxes from corn fields: on-farm assessment of the amount and timing of nitrogen fertilizer. Global Change Biol 16: 156–170.

[11] Rochette P, Tremblay N, Fallon E, Angers DA, Chantigny MH, MacDonald JD, et al (2010) N_2_O emissions from an irrigated and non-irrigated organic soil in eastern Canada as influenced by N fertilizer addition. Eur J Soil Sci 61: 186–196.

[12] Mosier AR, Kroeze C (2000) Potential impact on the global atmospheric N_2_O budget of the increased nitrogen input required to meet future global food demands. Chemosphere—Global Change Sci 2: 465–473.

[13] Kim D-G, Hernandez-Ramirez G, Giltrap D (2013) Linear and nonlinear dependency of direct nitrous oxide emissions on fertilizer nitrogen input: A meta-analysis. Agr Ecosyst Environ 168: 53–65.

[14] McSwiney CP, Robertson GP (2005) Nonlinear response of N_2_O flux to incremental fertilizer addition in a continuous maize (*Zea mays* L.) cropping system. Global Change Biol 11: 1712–1719.

[15] Kennedy TL, Suddick EC, Six J (2013) Reduced nitrous oxide emissions and increased yields in California tomato cropping systems under drip irrigation and fertigation. Agr Ecosyst Environ 170: 16–27.

[16] Van Groenigen JW, Velthof GL, Oenema O, Van Groenigen KJ, Van Kessel C (2010) Towards an agronomic assessment of N_2_O emissions: a case study for arable crops. Eur J Soil Sci 61: 903–913.

[17] Saarnio S, Heimonen K, Kettunen R (2013) Biochar addition indirectly affects N_2_O emissions via soil moisture and plant N uptake. Soil Biol Biochem 58: 99–106.

[18] Davidson EA (1991) Fluxes of nitrous oxide and nitric oxide from terrestrial ecosystems In: American Society for Microbiology, Washington DC pp. 219–235.

[19] Choudhary MA, Akramkhanov A, Saggar S (2002) Nitrous oxide emissions from a New Zealand cropped soil: tillage effects, spatial and seasonal variability. Agr Ecosyst Environ 93: 33–43.

[20] Dobbie KE, Smith KA (2003) Nitrous oxide emission factors for agricultural soils in Great Britain: the impact of soil water-filled pore space and other controlling variables. Global Change Biol 9: 204–218.

[21] Bhatia A, Sasmal S, Jain N, Pathak H, Kumar R, Singh A (2010) Mitigating nitrous oxide emission from soil under conventional and no-tillage in wheat using nitrification inhibitors. Agr Ecosyst Environ 136: 247–253.

[22] Parkin TB Kaspar TC (2006) Nitrous oxide emissions from corn-soybean systems in the Midwest. J Environ Qual 35: 1496–1506. 16825470 10.2134/jeq2005.0183

[23] Singurindy O, Molodovskaya M, Richards BK, Steenhuis TS (2009) Nitrous oxide emission at low temperatures from manure-amended soils under corn (*Zea mays* L.). Agr Ecosyst Environ 132: 74–81.

[24] Kloss S, Zehetner F, Dellantonio A, Hamid R, Ottner F, Liedtke V, et al (2012) Characterization of slow pyrolysis biochars: effects of feedstocks and pyrolysis temperature on biochar properties. J Environ Qual 41: 990–1000. 10.2134/jeq2011.0070 22751041

[25] Adviento-Borbe MAA, Haddix ML, Binder DL, Walters DT, Dobermann A (2007) Soil greenhouse gas fluxes and global warming potential in four high-yielding maize systems. Global Change Biol 13: 1972–1988.

[26] Cullum RF (2012) Influence of tillage on maize yield in soil with shallow fragipan. Soil Till Res. 119: 1–6.

[27] Lentz RD, Ippolito JA (2014) Biochar and manure affect calcareous soil and corn silage nutrient concentrations and uptake. J Environ Qual 41: 1033–1043. 10.2134/jeq2011.0126 22751045

[28] Venterea RT, Maharjan B, Dolan MS (2011) Fertilizer source and tillage effects on yield-scaled nitrous oxide emissions in a corn cropping system. J Environ Qual 40: 1521–1531. 10.2134/jeq2011.0039 21869514

[29] Sainju M, Stevens WB, Caesar-TonThat T, Liebig MA, Wang J (2014) Net global warming potential and greenhouse gas intensity influenced by irrigation, tillage, crop rotation, and nitrogen fertilization. J Environ Qual. 10.2134/jeq2013.10.0405 25602807

[30] Schellenberg DL, Alsina MM, Muhammad S, Stockert CM, Wolff MW, Sanden BL, et al (2012) Yield-scaled global warming potential from N_2_O emissions and CH_4_ oxidation for almond (*Prunus dulcis*) irrigated with nitrogen fertilizers on arid land. Agr Ecosyst Environ 155: 7–15.

[31] Smart DR, Alsina MM, Wolff MW, Matiasek MG, Schellenberg DL, Edstrom JP, et al (2011) N_2_O emissions and water management in California perennial crops In Guo L, Gunasekara AS, McConnell LL, editors. Understanding Greenhouse Gas Emissions from Agricultural Management, American Chemical Society, Baltimore MD USA pp. 227–255.

[32] Verhoeven E, Six J (2014) Biochar does not mitigate field-scale N_2_O emissions in a Northern California vineyard: An assessment across two years. Agr Ecosyst Environ 191: 27–38.

[33] Linquist B, Groenigen KJ, Adviento-Borbe MA, Pittelkow C, Kessel C (2011) An agronomic assessment of greenhouse gas emissions from major cereal crops. Global Change Biol 18: 194–209.

[34] DeFelice MS, Carter PR, Mitchell SB (2006) Influence of tillage on corn and soybean yield in the United States and Canada. Crop Manag. 10.1094/CM-2006-0626-01-RS

[35] Chen Y, Liu S, Li H, Li XF, Song CY, Cruse RM, et al (2011) Effects of conservation tillage on corn and soybean yield in the humid continental climate region of Northeast China. Soil Till Res. 115: 56–61.

[36] Franzluebbers AJ (2005) Soil organic carbon sequestration and agricultural greenhouse gas emissions in the southeastern USA. Soil Till Res 83: 120–147.

[37] Zheng J, Stewart CE, Cotrufo MF (2012) Biochar and nitrogen fertilizer alters soil nitrogen dynamics and greenhouse gas fluxes from two temperate soils. J Environ Qual 41: 1361–1370. 10.2134/jeq2012.0019 23099927

[38] Phillips RL, Tanaka DL, Archer DW, Hanson JD (2009) Fertilizer application timing influences greenhouse gas fluxes over a growing season. J Environ Qual 38: 1569–1579. 10.2134/jeq2008.0483 19549933

[39] Ussiri DAN, Lal R, Jarecki MK (2009) Nitrous oxide and methane emissions from long-term tillage under a continuous corn cropping system in Ohio. Soil Till Res 104: 247–255.

[40] Molodovskaya M, Warland J, Richards BK, Oberg G, Steenhuis TS (2011) Nitrous oxide from heterogeneous agricultural landscapes: source contribution analysis by eddy covariance and chambers. Soil Sci Soc Am J 75: 1829–1838.

[41] Huang HY, Wang JM, Hui DF, Miller DR, Bhattarai S, Jima T, et al (2014) Nitrous oxide emission from a commercial cornfield (*Zea mays*) measured using the eddy-covariance technique. Atmos Chem Phys Discussion, 14: 20417–20460.

[42] Aulakh MS, Rennie DA, Paul EA (1984) Gaseous nitrogen losses from soils under zero-till as compared with conventional-till management systems. J Environ Qual 13: 130–136.

[43] Franzluebbers AJ, Hons FM, Zuberer AD (1995) Tillage and crop effects on seasonal soil carbon and nitrogen dynamics. Soil Sci Soc Am J 59: 1618–1624.

[44] Neftel A, Flechard CR, Ammann C, Conen F, Emmenegger L, Zeyer K (2007) Experimental assessment of N_2_O background fluxes in grassland systems. Tellus 59B: 470–482.

[45] Menneer JC, Ledgard SF, Sprosen MS (2008) Soil N process inhibitors alter nitrogen leaching dynamics in a pumice soil. Aust. J Soil Res 46: 323–331.

[46] Kelliher FM, Clough TJ, Clark H, Rys G, Sedcole JR (2008) The temperature dependence of dicyandiamide (DCD) degradation in soils: A data synthesis. Soil Biol Biochem 40: 1878–1882.

[47] Downie A, Crosky A, Munroe P (2009) Physical properties of biochar In: Lehmann L, Joseph S, editors. Biochar for environmental management: Science and technology. Earthscan, London pp. 13–32.

[48] Cayuela ML, van Zwieten L, Singh BP, Jeffery S, Roig A, Sánchez-Monedero MA (2013) Biochar's role in mitigating soil nitrous oxide emissions: A review and meta-analysis. Agr Ecosyst Environ 191: 5–16.

[49] Asai H, Samson BK, Stephan HM, Songyikhangsuthor K, Homma K, Kiyono Y, et al (2009) Biochar amendment techniques for upland rice production in Northern Laos 1. Soil physical properties, leaf SPAD and grain yield. Field Crops Res 111: 81–84.

[50] Van Zwieten L, Kimber S, Morris S, Chan YK, Downie A, Rust J, et al (2010) Effects of biochar from slow pyrolysis of papermill waste on agronomic performance and soil fertility. Plant Soil 37: 235–246.

